# MARCH8 is associated with poor prognosis in non-small cell lung cancers patients

**DOI:** 10.18632/oncotarget.22602

**Published:** 2017-11-21

**Authors:** Jiye Fan, Liying Tian, Manli Li, Shu-Hong Huang, Jing Zhang, Baohua Zhao

**Affiliations:** ^1^ Department of Biochemistry and Molecular Biology, College of Life Science, Hebei Normal University, Shijiazhuang, Hebei 050024, PR China; ^2^ Department of Pharmacy, Hebei Chemical and Pharmaceutical College, Shijiazhuang, Hebei 050026, PR China; ^3^ Department of Neurobiology, Shandong Provincial Key Laboratory of Mental Disorders, School of Basic Medical Science, Shandong University, Jinan, Shandong 250012, PR China

**Keywords:** MARCH8, ubiquitin ligase, NSCLC, proliferation, apoptosis

## Abstract

MARCH8 belongs to a family of membrane-associated RING-CH (MARCH) ubiquitin ligases. The functions of MARCH8 have been thoroughly investigated but its mechanism of action remains unknown. In this study, we detected the expression of MARCH8 protein in NSCLC samples and identified MARCH8 mRNA expression through a TCGA database. In addition, we analyzed the correlation between MARCH8 and the clinical characteristics of NSCLC patients and their prognosis.(www.kmplot.com). The roles of MARCH8 in proliferation, migration, and metastasis were further explored through ectopic expression analysis and western blot analysis; its mechanism of expressionwas also explored. We discovered that MARCH8 was downregulated in NSCLC tissues compared to adjacent normal lung tissues. Overexpression of MARCH8 inhibited NSCLC cell proliferation and metastasis via the PI3K and mTOR signaling pathways; this also increased apoptosis of A549 and H1299 cells. Our results indicated that MARCH8 plays crucial roles in NSCLC against carcinogenesis and progression; therefore, MARCH8 might be a predictive factor and an attractive therapeutic target for NSCLC patients.

## INTRODUCTION

Lung cancer involves malignant tumors and is very harmful to humans. More than 80 percent of lung cancers are classified as non-small cell lung cancer (NSCLC)[1]. Unfortunately, even though treatment modalities have improved over time, the 5-year survival rate is still less than 15 percent. This is mainly due to relapse and metastasis [2]. Investigation of novel prognostic biomarkers and the development of new therapeutic targets are of great importance.

Membrane-associated RING-CH 8 (MARCH8) is one of the members of the recently discovered MARCH family of RING-finger E3 ubiquitin ligases [3]. MARCH8 has been reported to downregulate several proteins, such as major histocompatibility complex (MHC)-II, CD86, interleukin (IL)-1 receptor accessory protein, TNF-related apoptosis-inducing ligand (TRAIL) receptor 1 and the transferrin receptor [3–8]. However, the physiological roles of MARCH8 in lung cancer remain largely unknown. In this study, we sought to determine the crucial roles MARCH8 plays in NSCLC against carcinogenesis and its progression.

## RESULTS

### Expression level of MARCH family in NSCLC

**Figure 1:**
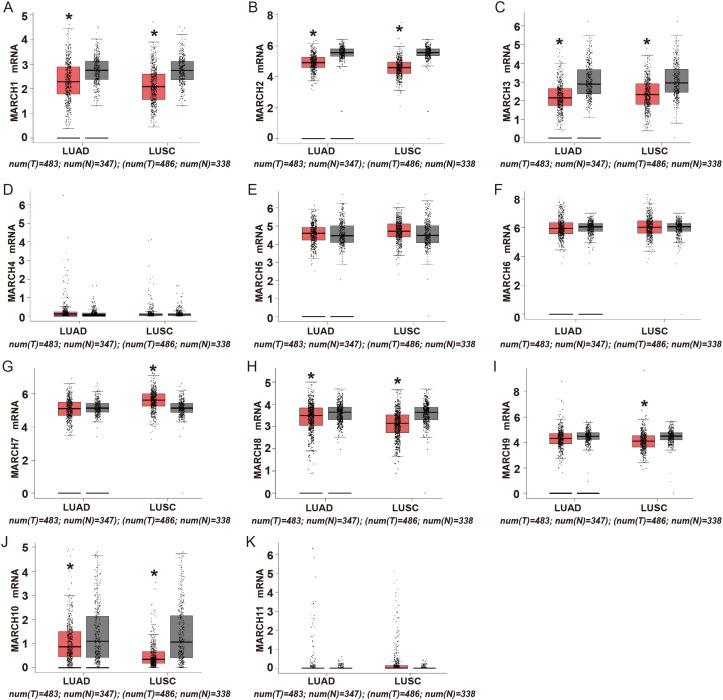
Expression of MARCH family members in human lung cancer **(A-K)** Analysis of MARCH family members in TCGA database. The red and gray boxes represent the normal and cancerous tissues, respectively (^*^p<0.05).

Members of the MARCH family have a wide variety of cell functions, including immune regulation, membrane transport and endoplasmic reticulum-associated degradation (ERAD). We first analyzed the expression of the MARCH family using Gene Expression Profiling Interactive Analysis (GEPIA), which is an online tool for transcriptomic analysis based on the Cancer Genome Atlas (TCGA) and the Genotype-Tissue Expression (GTEx) dataset [9]. As shown in Figure 1, the expression of MARCH1, MARCH2, MARCH3, MARCH8 and MARCH10 decreased significantly in lung adenocarcinoma (LUAD) and lung squamous cell carcinoma (LUSC) compared to normal lung tissue, and MARCH9 mRNA level decreased significantly in LUSC. These results demonstrated that most members of the MARCH family, including MARCH8, were down-regulated in human lung cancer. This suggests that members of this family have potential antitumor effects.

### Expression level of MARCH8 in NSCLC tissues

**Figure 2:**
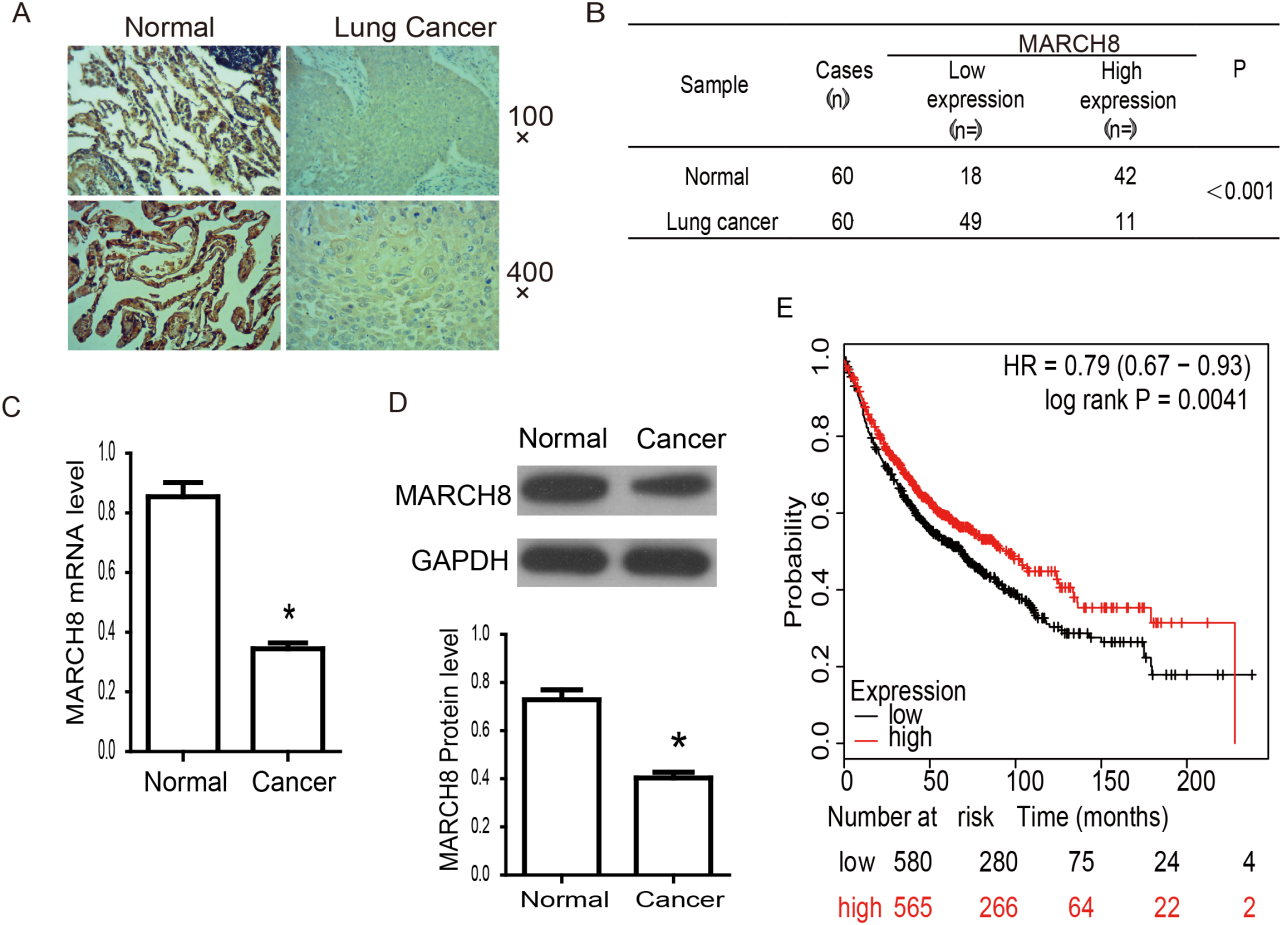
MARCH8 expression and association with prognosis in human lung cancer **(A)** The expression of MARCH8 was detected by Immunohistochemistry in human normal and lung cancer tissues. Representative images were shown. There was strong brown staining in normal lung tissues and much lighter staining in cancer tissues. **(B)** Analysis of Immunohistochemistry results. MARCH8 is significantly down-regulated in lung cancer tissues (*p*<0.001). **(C)** MARCH8 mRNA levelin human normal and lung cancer tissues was confirmed by qPCR. MARCH8 mRNA level in lung cancer tissuesdecreased significantly compared with that in normal tissues. **(D)** MARCH8 protein level in human normal and lung cancer tissues was confirmed by western blot. MARCH8 protein level in lung cancer tissuesdecreased significantly compared with that in normal tissues. **(E)** Overall survival analysis. High expression of MARCH8 was found to be associated with better prognosis in lung cancer.

The down-regulated expression of MARCH8 demonstrates the potential importance in the prognostic implications of NSCLC. To evaluate this, we first detected the expression level of MARCH8 in tissue sections by using immunohistochemistry (Figure 2A and 2B). MARCH8 showed strong expression in control normal tissues, while expression was significantly decreased in cancer cells (Figure 2). Low-level expression of MARCH8 was observed in most of the lung cancer samples (49 of 60, 81.67%), whereas a much lower percentage of normal tissue showed low expression of MARCH8 (18 of 60, 30%). We further detected the mRNA and protein level of MARCH8 on human lung cancer and normal tissues using q RT-PCR and western blot analysis (Figure 2C and 2D). MARCH8 was down-regulated in human lung cancer tissues in comparison to normal tissues. Because the expression of MARCH8 was significantly different in cancer and normal tissues, we next investigated the association between MARCH8 expression level and survival rates of lung cancer patients. The data from the Kaplan-Meier Plotter database was analyzed by *in silico* analysis (http://kmplot.com/analysis/index.php?p=service&start=1). The results revealed that the high expression of MARCH8 was positively correlated with significant improvement in patient survival rates in NSCLC (Figure 2E).

### MARCH8 inhibits the proliferation of A549 and H1299 cells

**Figure 3:**
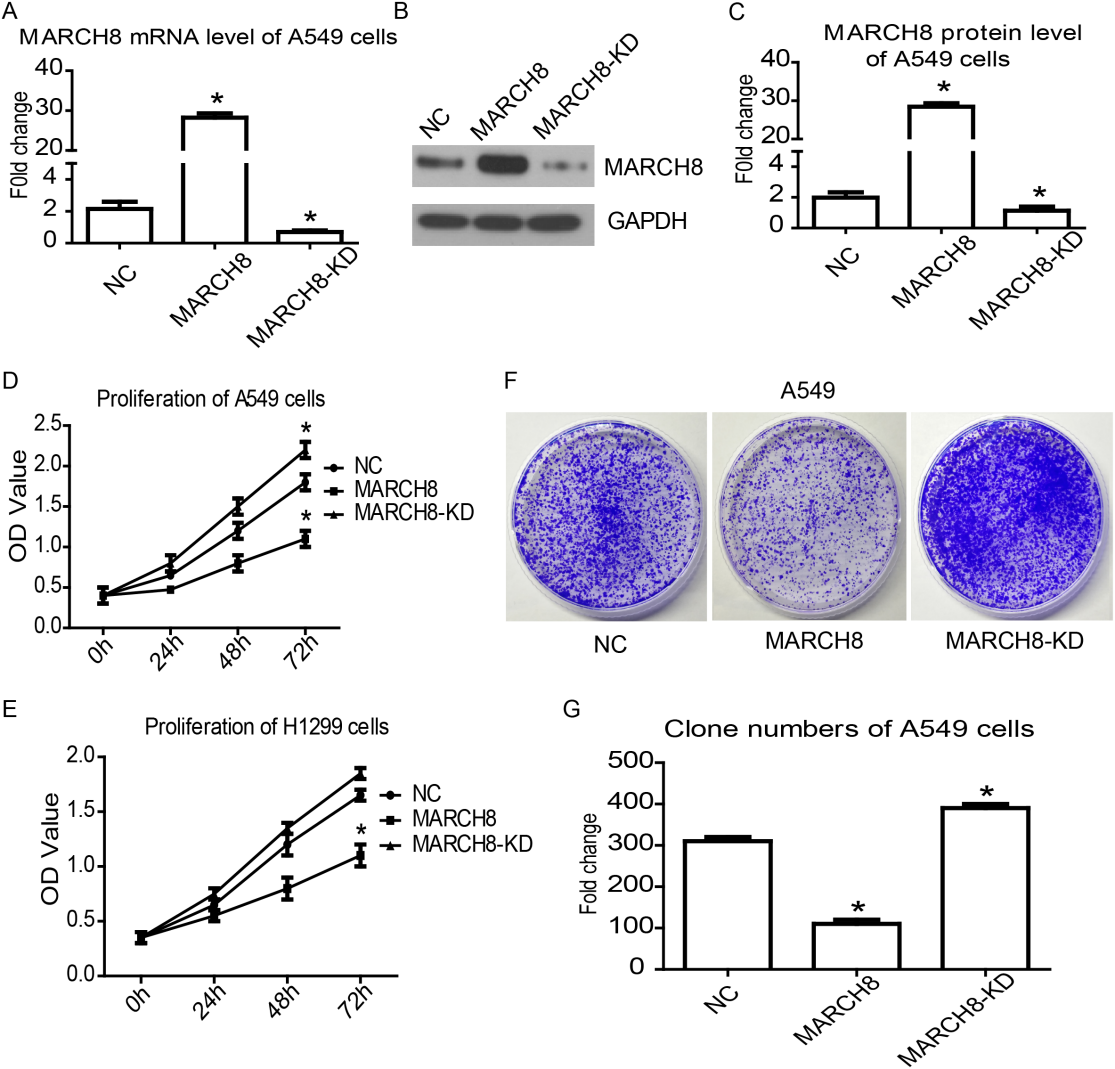
MARCH8 inhibits cancer cell proliferation in human lung cancer cells Quantitative RT-PCR **(A)** and western blot (B) were used to detect MARCH8 expression level in A549 cell line transferred with MARCH8-overexpression vector (MARCH8), MARCH8-siRNA (MARCH8-KD) and blank vector (NC). The representative results of western blot analysis and bands intensity analysis in column chart were shown **(B** and **C)**. Cell counting kit-8 assay was performed to detect cell proliferation in A549 **(D)** and H1299 **(E)** cell lines. Colony formation assay **(F)** was performed to further confirmcell proliferation in the A549 cell line, and analysis of colony formation assay was shown **(G)**.

To investigate the tumor suppressor function of MARCH8, we overexpressed exogenous MARCH8 in NSCLC cell lines A549 and H1299 by transiently transfecting a vector containing MARCH8. Meanwhile, a siRNA oligonucleotide targeting MARCH8 was used to inhibit endogenous MARCH8 expression. The cell proliferation was analyzed *in vitro* by CCK8 assay. The increased mRNA and protein expression of MARCH8 from transfected DNA was confirmed by qRT-PCR and western blot analysis (Figure 3A-3C). The CCK8 assay results showed that the proliferation of A549 and H1299 cells were significantly inhibited by overexpression of MARCH8. Furthermore, inhibition of MARCH8 promoted proliferation of these cells (Figure 3D and 3E). The colony formation assay confirmed that the colony-forming activity was lower in the MARCH8 group, but higherin the MARCH8-KD group, when compared to the negative control group (Figure 3F and 3G). This data indicated that MARCH8 can inhibit the proliferation of human lung cancer cells.

### MARCH8 inhibits the migration and invasion of A549 cells

**Figure 4:**
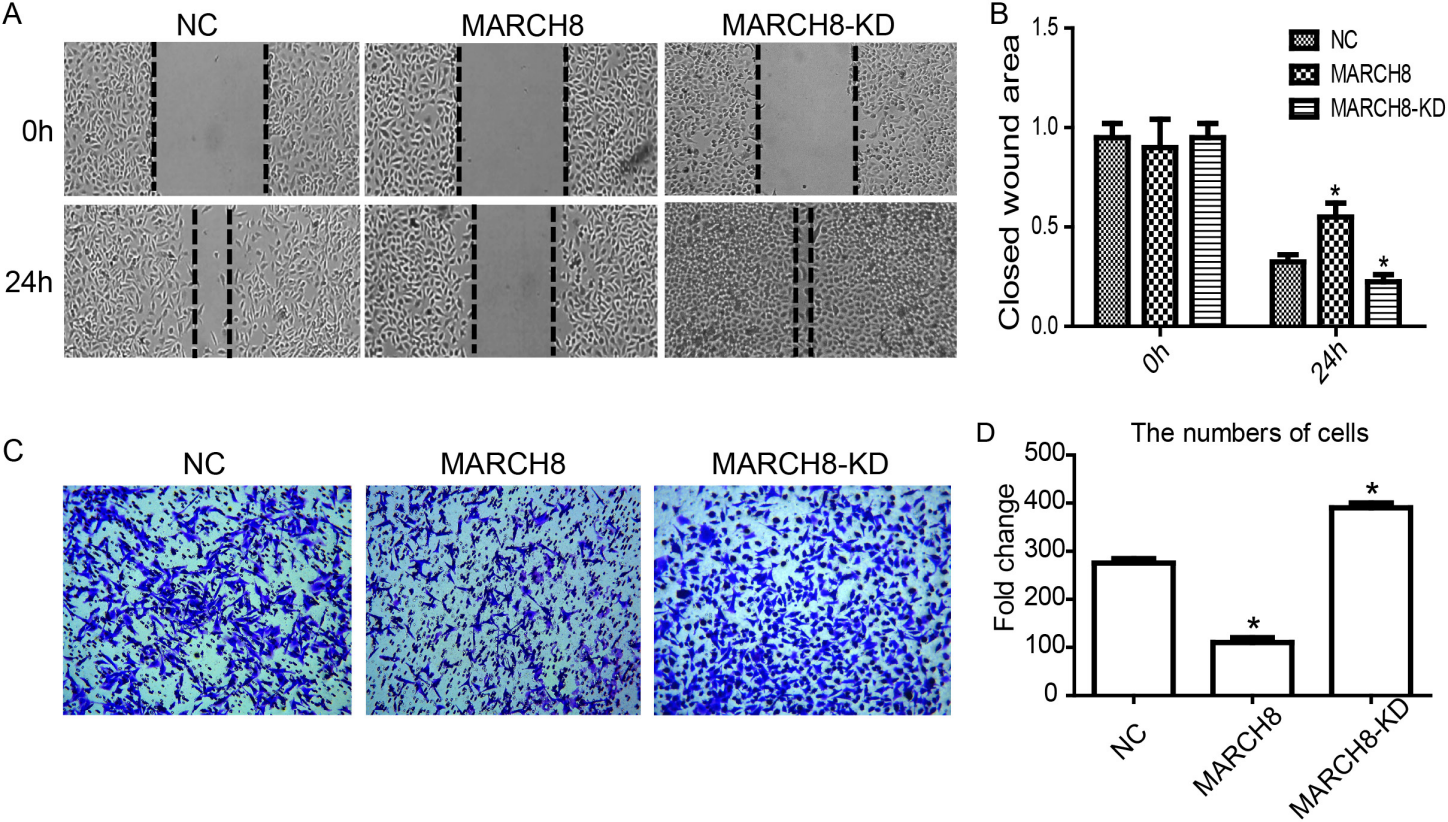
MARCH8 inhibits migration and invasion of lung cancer cell A549 *in vitro* **(A)** Wound healing assay. **(B)** Wound area of 0h and 24h comparison. **(C)** Trans-well assay. **(D)** Migrated cells were pictured from five random fields to analyze the invasion activity.

To evaluate whether MARCH8 is involved in tumor migration and metastasis, we performed wound healing and trans-well assays to investigate the effect of MARCH8 on the migratory and invasive potential of A549 cells. The wound healing assay revealed that the migratory ability of A549 cells was significantly reduced in the MARCH8 group and increased in the MARCH8-KD group 48 hours after wounding compared with the respective control groups (Figure 4A and 4B, p< 0.05). The cell invasion assay showed that in A549 cells the number of invading cells was significantly lower in the MARCH8 group and significantly higher in the MARCH8-KD group than in the control group (Figure 3C and 3D, p< 0.05). These results suggest that MARCH8 has a functional role in cell migration and metastasis of NSCLC.

### MARCH8 promotes apoptosis of NSCLC cells

**Figure 5:**
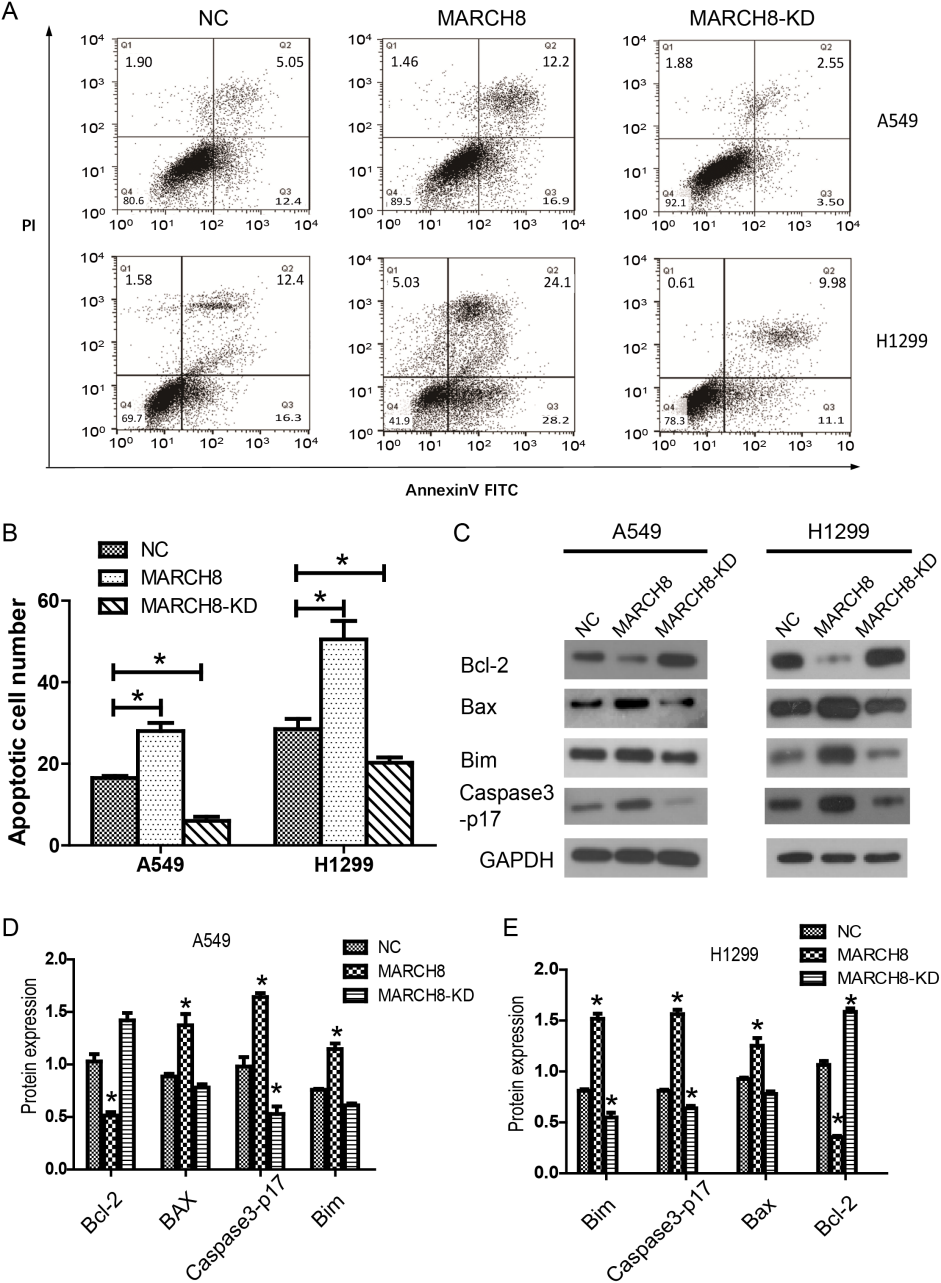
MARCH8 promoted apoptosis of lung cancer cells *in vitro* **(A)** Apoptosis of the cells was determined by flow cytometry. **(B)** The apoptotic cell ratio was shown in the column chart, in which the MARCH8 group was significantly higher, while the MARCH8-KD group was significantlylower than the NC group. **(C)** Apoptotic activity was measured by Bcl-2, Bax, Bim and Caspase3-p17 activity. Quantitative results of A549 **(D)** and H1299 **(E)** are shown in the panelbelow. The error bars indicate ± SEM. ^*^P<0.05 by Student's t-test.

To investigate whether MARCH8 plays a role in NSCLC growth through apoptotic regulation, fluorescent-activated cell sorting (FACS) was performed to detect Annexin V and Propidium Iodide (PI) positive cells in MARCH8 and MARCH8-KD groups of A549 and H1299 cells. Compared with non-transfected cells, the knockdown of MARCH8 reduced the apoptotic cell number. Meanwhile, MARCH8 overexpression dramatically increased Annexin V-positive cell numbers, indicating that MARCH8 overexpression induced apoptosis of A549 cells (Figure 5A and 5B). To investigate the molecular mechanism by which MARCH8 induced apoptosis in A549 and H1299 cells, we detected theexpression of apoptosis related genes in each group. We found that expression levels of cleaved caspase-3, as well as pro-apoptotic proteins Bax and Bim, were significantly increased while anti-apoptotic protein Bcl-2 was down-regulated in MARCH8 overexpressed A549 and H1299 cells. Similarly, expression levels of cleaved caspase-3, Bax, and Bim were reduced while Bcl-2 was increased in MARCH8 knockdown cells (Figure 5C-5E). These results indicated that MARCH8 may induce apoptosis of A549 cells.

### MARCH8 blocks the activation of the AKT pathways and causes the reversal of EMT

**Figure 6:**
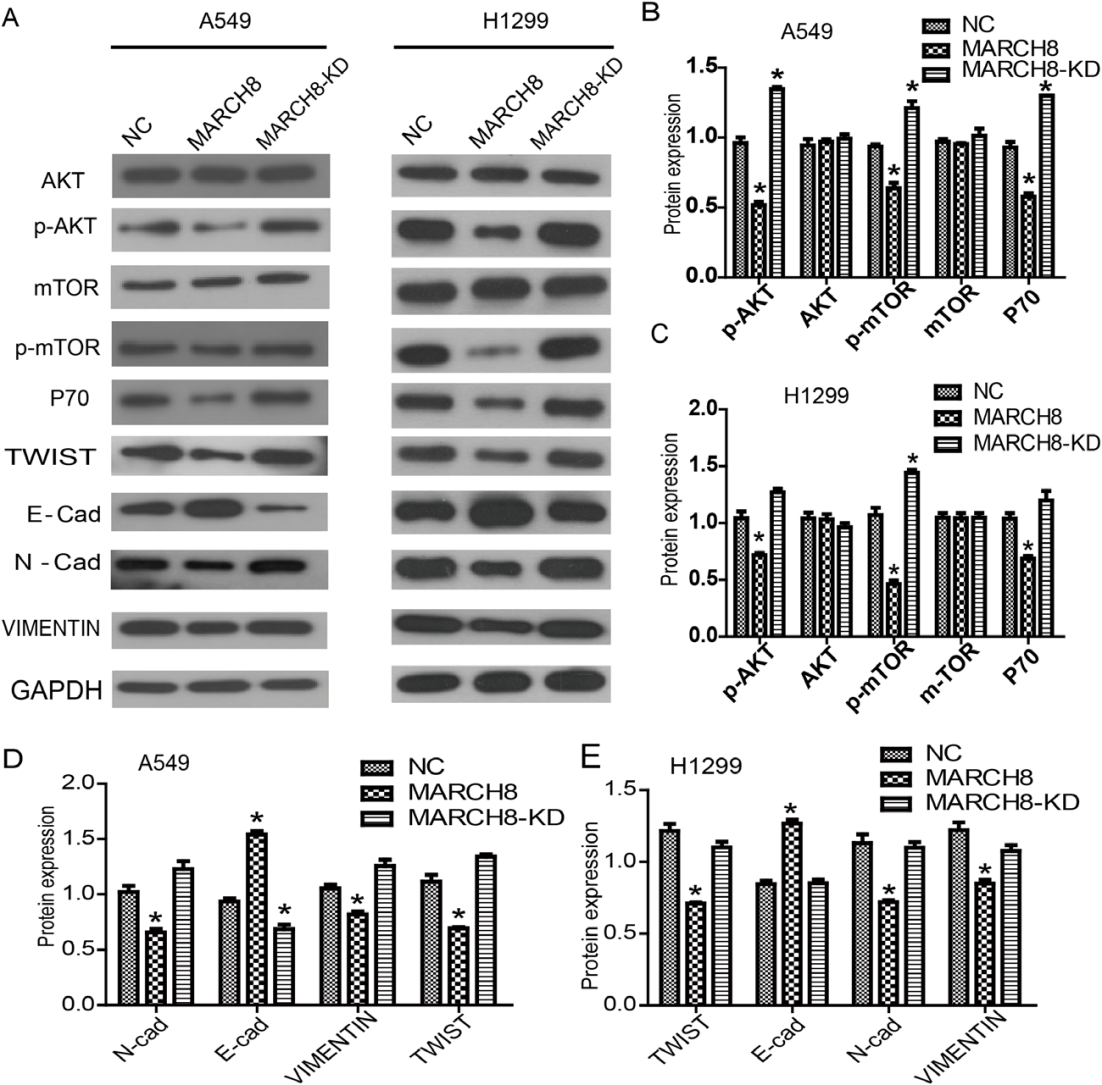
MARCH8 was involved in PI3K/AKT signaling pathway and epithelial-mesenchymal transition (EMT) process **(A)** PI3K/AKT signaling related genes were down-regulated in the MARCH8 group, as well as the downstream genes related with cell proliferation. Meanwhile, overexpression of MARCH8 inhibited the expression of EMT related genes. Quantitative results of PI3K/AKT signaling related genes in A549 **(B)** and H1299 cells **(C)** are shown. The error bars indicate ± SEM. ^*^P<0.05 by Student's t-test. Quantitative results of EMT related genes in A549 **(D)** and H1299 cells **(E)** are shown. The error bars indicate ± SEM. ^*^P<0.05 by Student's t-test.

**Figure 7:**
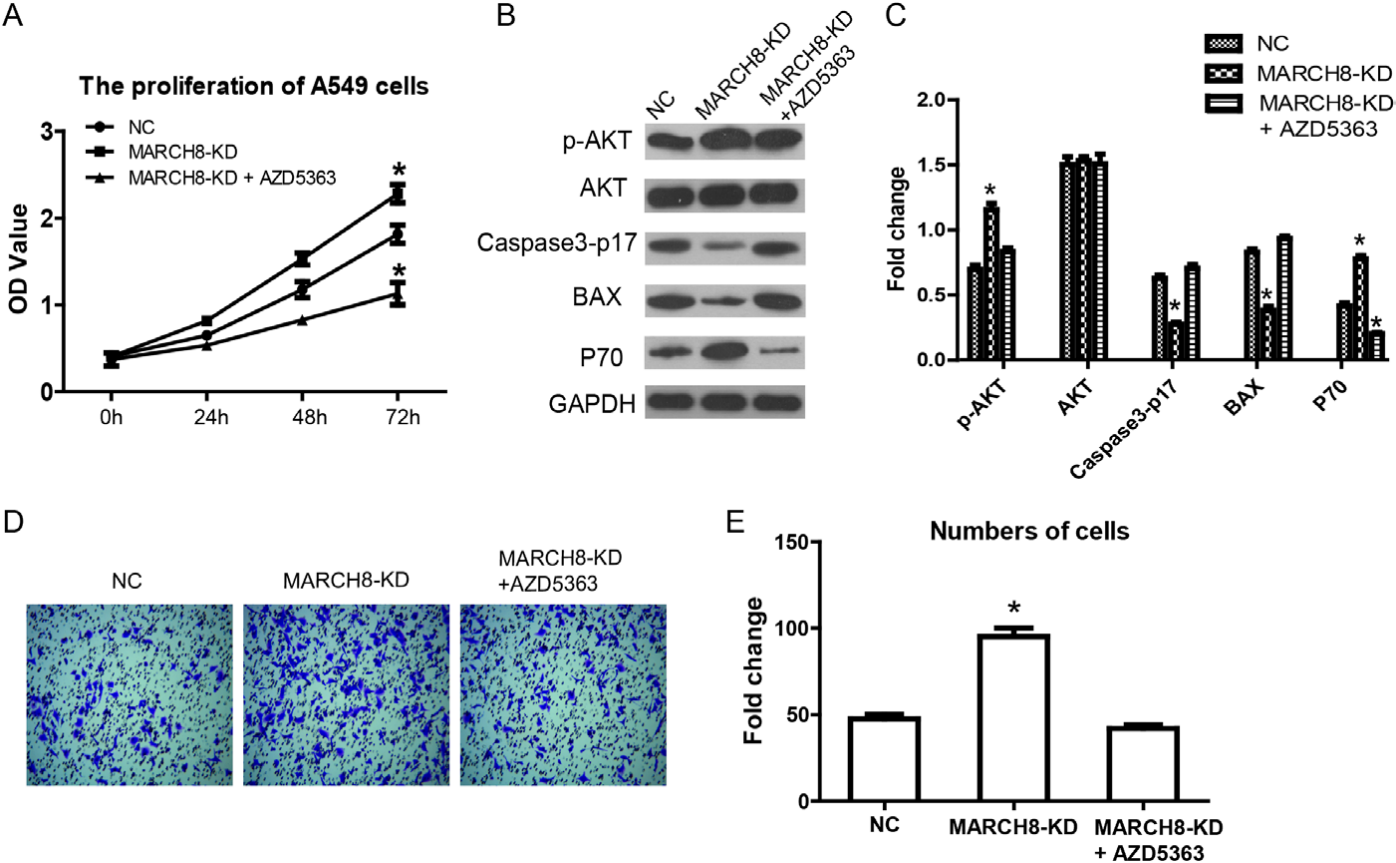
AKT inhibitor blocks the effect of MARCH8 in human lung cancer cells AKT inhibitor, AZD5363, was used in singling inhibitor experiment. **(A)** Cell counting kit-8 assay was performed to detect cell proliferation inMarch8-KD group and March8-KD+ AZD5363. **(B)** AKT signaling and proliferation related genes were detected in A549 cells. **(C)** Quantitative results are shown. The error bars indicate ± SEM. ^*^P<0.05 by Student's t-test. **(D)** The effect of March8 on cell invasion was blocked by AZD5363 in A549 cells. **(E)** Quantitative resultsare shown. The error bars indicate ± SEM. ^*^P<0.05 by Student's t-test.

Then, we investigated the mechanism by which MARCH8 inhibits tumorigenesis. According to present knowledge, PI3K/AKT/mTOR is a crucial signaling pathway in tumorigenesis, including human lung cancer. Therefore, we detected the levels of signaling-related genes in cells of a MARCH8 overexpression group and a knockdown group. We found that MARCH8 overexpression significantly decreased the protein levels of p-AKT and p-mTOR without affecting the total protein levels; the MARCH8 knockdown group increased the protein levels of p-AKT and p-mTOR both in A549 and A1299 cells (Figure 6), suggesting the involvement of MARCH8 in PI3K-AKT-mTOR signaling. What is more, we found AKT inhibitor, AZD5363, could blocks the effect of MARCH8 in A549 cells (Figure 7). We then detected the EMT related protein, which is closely related to tumor progression. Transfection of the cells with a MARCH8 vector increased E-cadherin expression and decreased N-cadherin, Snail and Twist expression. Opposite results were obtained in the MARCH8 knockdown group (Figure 6). The change in expression of EMT-associated proteins indicated that overexpression of MARCH8 reversed EMT.

## DISCUSSION

Extensive studies have revealed that ubiquitination is a highly important process for post-translational regulation of proteins [10]. In regards to cancer, ubiquitination plays different roles which depends on the type and status of progression [10].

The RING E3 groups is one of the main classes of E3 ligases [11]. MARCH (Membrane-Associated RING-CH) proteins belong to RING E3s but have a RING-CH domain, which differs from the classic RING domain (now termed as RING-HC)[12]. The first identified mammalian MARCH protein is MARCH8, originally termed c-MIR [12]. Since the discovery of MARCH8, ten more MARCH proteins were identified. These proteins make up an eleven members family, in which the E3 ubiquitin ligase activity lies in the common RING-CH domain [13].

MARCH8 is expressed in many human tissues and cell types, including neonatal brain, lymph node, spleen, placenta, heart, liver, kidney and lung. The highest level of expression is in the lung tissue. In cells, MARCH8 was found in early and late endosomes as well as on the cell surface [13].

Overexpression of MARCH8 leads to downregulation of several immunomodulatory receptors, such as MHC I, HLA 2.1, and MHC II, indicating that it plays a significant role in immune suppression [12, 14–18]. MARCH8 down-regulates TNF-related apoptosis inducing ligand receptor 1 (TRAIL-R1; also known as DR4) on the surface of breast cancer cells, but has no effect on TRAIL-R2 [19]. Overall, the expression of MARCH8 prevents cells from undergoing apoptosis. This indicates that targeting MARCH8 for knockdown may provide therapeutic benefits to patients with cancer [19]. Furthermore, MARCH8 has been identified in a siRNA screen aimed to isolate restriction factors to HIV-1 replication [20].

Overexpression of MARCH8 leads to the downregulation of surface TfR, indicating that this is another method of regulation for surface TfR in HepG2 cells [21]. To date, studies of the MARCH proteins have focused on their function in the immune system.

Our results provide several new insights regarding the mechanism and significance of MARCH8-related NSCLC. We found that the expression of MARCH8 was decreased in NSCLCfor the first time. We showed that MARCH8 acted as atumor-suppressing gene and inhibited many cell functions of A549 and H1299 cells. MARCH8 inhibited cancer cell proliferation, migration and invasion of NSCLC. We found that overexpression of MARCH8 could reverse EMT by affecting expression of EMT-associated proteins, which suggest MARCH8 is involved in the PI3K-AKT-mTOR signaling pathway.

Currently, there are few studies of MARCH8 in tumors. We hypothesized that the low expression of MARCH8 in lung cancer could be due to the regulation of aberrant transcription factors or epigenetic regulation, which may also be a key factor in the role of MARCH8 in human lung cancer. However, there is no evidence to support this, and this will be the focus of our future research. Through in-depth studies on the regulatory mechanism, we expectto excavatethe clinical value of MARCH8 as a clinical indicator and potential therapeutic target.

In summary, we have demonstrated in this study that the expression of MARCH8 was decreased in human NSCLC samples, suggesting MARCH8 could serve as a biomarker for NSCLC early diagnosis. As an E3 ubiquitin ligase, the target of MARCH8 in NSCLC, and the signaling pathway mediated by MARCH8 still need to be further investigated.

## MATERIALS AND METHODS

### Cell culture and transfection

The human lung adenocarcinoma cell lines A549 and H1299 were purchased from the Type Culture Collection of the Chinese Academy of Sciences (Shanghai, China). Cells were cultured in Dulbecco's modified Eagle's medium (DMEM) (Gibco, Carlsbad, CA, USA) containing 10% fetal bovine serum (Gibco), 100 IU/mL penicillin, and 100 μg/mL streptomycin (Invitrogen, CA, USA) at 37°C in a humidified atmosphere of 5% CO_2_. Transient transfection was performed using Lipofectamine 2000 (Invitrogen) following the standard protocol.

### Cell growth and proliferation

Cell Counting Kit-8 (CCK-8) was used to assess the effects of MARCH8 on the viability of A549 and H1299 cells in different groups (negative control, and MARCH8 overexpression groups). In brief, the CCK-8 reagent was added to each treated group of wells with 1:10 (v/v) per 100 μl of medium when cells were transfected for 0, 24, 48, and 72 hours. After incubation, optical density (OD) at 450 nm was determined for the supernatant of each well by a microplate reader. Data was collected from at least three independent experiments, each time in triplicate.

### Flow cytometry (FCM) detection

Apoptotic cells in two groups were identified by flow cytometry and the Annexin-V/Fluorescein Isothiocyanate (FITC) kit (BD Biosciences). A549 cells were collected and re-suspended, followed by incubation in 200 μl of binding buffer containing 5 μl of Annexin-V/FITC at room temperature in the dark for 10 minutes. After being centrifuged at 1,000g for 5 minutes at room temperature, the supernatant was discarded and the cells were re-suspended in 200 μl of fresh binding buffer. Then, 10 μl of PI was gently added and incubated on ice in the dark for two minutes. Finally, 400 μl of PBS was added and flow cytometric analyses were performed.

### RNA isolation and quantitative real-time PCR

The total RNA of cells was extracted by using an Ultrapure RNA Kit (CWbio, Beijing China) according to the manufacturer's protocol. Reverse transcription was performed through use of a First Strand cDNA Synthesis Kit (Qiagen, Hilden, Germany) following the manufacturer's instructions. The relative mRNA level of MARCH8 was determined by quantitative real-time PCR (qRT-PCR) by using the UltraSYBR Mixture (CWbio). Quantitative RT-PCR was performed in a cycler (Light Cycler 2.0; Roche). The relative expression level of MARCH8 mRNA was calculated by the 2^-ΔCt^ method and normalized to GAPDH. All primers were synthesized by GeneWiz (Beijing, China) as follows: MARCH8–F, 5’-GGGAGAAGTTGCAGATGAC-3’; MARCH8–R, 5’-GCACATACAAGGACCAGAC-3’; GAPDH–F, 5’-CGGAGTCAACGGATTTGGTCGTAT-3’; GAPDH–R, 5’-AGCCTTCTCCATGGTGGTGAAGAC-3’. Each sample was performed in triplicate.

### Colony formation assay

Cells were grown on a 6-well plate at an initial density of 100 cells per well at 37°C for 14 days with the culture medium changing every 2 days. Cell colonies were fixed with 4% paraformaldehyde for 10 minutes and stained with crystal violet for 20 minutes. After counting, cell colonies were photographed.

### Wound healing assay

Cells were seeded into a 6-well plate (2×10^5^ cells per well) and cultured for 24 hours in normal medium before scratching. A sterile 200 μl pipette tip was used to scratch a straight line through the cell layer in each well. After incubation in low serum (2%) for 48 hours under standard conditions, the wound closure was quantified by measuring the remaining un-migrated area with Image J. Assays were performed three times.

### Cell invasion assay

The 8-μm pore size Trans-well inserts(Sigma-Aldrich, San Francisco, CA, USA) and the 24-well plate were washed with PBS for 5 minutes before the experiment. The inserts were coated with 200μl of Matrigel (dilution at 1:2; BD Biosciences). Cells in 0.5 ml serum-free medium at a density of 1×10^5^ cells/ml were transferred to the upper Matrigel chambers with 0.75 ml of 10% serum complete medium as the chemoattractant in the lower chamber and incubated at 37°C for 48 hours. Cells that were able to pass through the filter were fixed and stained with 0.5% crystal violet for 30 minutes. Finally, the numbers of invaded cells in five randomly selected high-power fields were counted under the microscope.

### Western blot analysis

The relationship between MARCH8 and tumor-regulated genes was determined through western blot analysis. Cells were harvested 48 hours after transfection, washed twice with PBS and lysed by radio immunoprecipitation assay buffer (RIPA; Beyotime, Shanghai, China) containing 0.01% protease and phosphatase inhibitor (Sigma-Aldrich, Shanghai, China) on ice for 30 minutes. The cell lysate was centrifuged at 12,000g for 10 minutes at 4°C. Proteins in the supernatant were collected and quantified by BCA assay. About 20–30 μg of protein samples in each load were separated by 10% SDS-PAGE gel and then transferred to a polyvinylidene fluoride membrane (Millipore, Shanghai, China). The membrane was blocked with 5% BSA in PBST and incubated with indicated primary antibodies overnight at 4°C. The membranes were rinsed with TBST buffer (0.1% Tween 20, 0.2 mMTris, and 137 mMNaCl) and incubated with HRP-conjugated secondary antibody (1:5000) for one hour at room temperature, followed by chemiluminescent detection.

### Statistical analysis

All statistical analyses were conducted by using the SPSS 19 software. Data was analyzed with student's t -tests. A value of *p*< 0.05 was considered statistically significant in all statistical comparisons.
